# Flavonoid biosynthesis is differentially altered in detached and attached ripening bilberries in response to spectral light quality

**DOI:** 10.3389/fpls.2022.969934

**Published:** 2022-07-22

**Authors:** Amos Samkumar, Katja Karppinen, Tony K. McGhie, Richard V. Espley, Inger Martinussen, Laura Jaakola

**Affiliations:** ^1^Department of Arctic and Marine Biology, UiT The Arctic University of Norway, Tromsø, Norway; ^2^The New Zealand Institute for Plant and Food Research Ltd., Palmerston North, New Zealand; ^3^The New Zealand Institute for Plant and Food Research Ltd., Auckland, New Zealand; ^4^Department of Horticulture, Norwegian Institute of Bioeconomy Research, Ås, Norway

**Keywords:** anthocyanins, flavonoids, flavonols, polyphenols, fruit ripening, light quality, *Vaccinium myrtillus* L.

## Abstract

Light spectral quality is known to affect flavonoid biosynthesis during fruit ripening. However, the response of fruits to different light conditions, when ripening autonomously from the parent plant (detached), has been less explored. In this study, we analyzed the effect of light quality on detached and naturally ripening (attached) non-climacteric wild bilberry (*Vaccinium myrtillus* L.) fruits accumulating high amounts of anthocyanins and flavonols. Our results indicated contrasting responses for the accumulation of phenolic compounds in the berries in response to red and blue light treatments. For detached berries, supplemental blue light resulted in the highest accumulation of anthocyanins, while naturally ripening berries had elevated accumulation under supplemental red light treatment. Both red and blue supplemental light increased the expression levels of all the major structural genes of the flavonoid pathway during ripening. Notably, the key regulatory gene of anthocyanin biosynthesis, *VmMYBA1*, was found to express fivefold higher under blue light treatment in the detached berries compared to the control. The red light treatment of naturally ripening berries selectively increased the delphinidin branch of anthocyanins, whereas in detached berries, blue light increased other anthocyanin classes along with delphinidins. In addition, red and far-red light had a positive influence on the accumulation of flavonols, especially quercetin and myricetin glycoside derivatives, in both ripening conditions. Our results of differential light effects on attached and detached berries, which lacks signaling from the mother plant, provide new insights in understanding the light-mediated regulatory mechanisms in non-climacteric fruit ripening.

## Introduction

Fruit ripening is a complex process associated with determining various quality attributes, such as firmness, flavor, aroma, and color development. Anthocyanin pigments, as responsible for color development, accumulate at high levels during ripening providing distinct red or blue coloration to many fruits (Zifkin et al., 2012; Oh et al., 2018). Although anthocyanins mostly contribute to the phenolic composition in red- and blue-colored fruits, flavonols, such as quercetin and myricetin, also contribute to the flavonoid content in many edible berries (Häkkinen et al., 1999; Wang et al., 2014). Both anthocyanins and flavonols are synthesized *via* the flavonoid branch from the well-characterized phenylpropanoid biosynthetic pathway. The key enzymes involved in flavonoid biosynthesis are chalcone synthase (CHS), along with flavonoid hydroxylases (F3'H and F3'5'H), which are the rate-limiting enzymes in the cyanidin and delphinidin branch-points, respectively. Flavonols are synthesized from dihydroflavonols by flavonol synthase (FLS). The late anthocyanin biosynthetic enzymes, such as dihydroflavonol 4-reductase (DFR) and anthocyanidin synthase (ANS), are involved in the production of anthocyanidin aglycons. UDP-glucose flavonoid 3-*O*-glucosyltransferase (UFGT) performs the glycosylation steps to the 3-hydroxyl group of anthocyanidins (Zhai et al., 2014). This sequential process leads to the production of different anthocyanin classes, including derivatives of delphinidins, cyanidins, pelargonidins, malvidins, petunidins, and peonidins (Dare et al., 2022).

Anthocyanin biosynthesis is regulated by the MBW complex, consisting of R2R3 MYB, basic helix–loop–helix (bHLH) and WD-40 repeat proteins, activating the key flavonoid structural genes (Petroni and Tonelli, 2011). In particular, the R2R3 MYB transcription factors have been shown to directly interact with promoters of the major flavonoid biosynthetic genes including *DFR*, *ANS* and *UFGT* (Plunkett et al., 2018; Die et al., 2020). Recent studies have identified specific transcription factors, *MYBA1* and *MYBPA1.1*, from the large R2R3 MYB family as the key regulators in *Vaccinium* berries. It has been demonstrated that these two MYBs co-regulate and promote anthocyanin biosynthesis during berry ripening (Plunkett et al., 2018; Die et al., 2020; Karppinen et al., 2021; Lafferty et al., 2022).

Light is known to be one of the major environmental factors controlling fruit ripening and anthocyanin biosynthesis (Ouzounis et al., 2015; Abeysinghe et al., 2019). Certain wavelengths from the photosynthetically active radiation (PAR) range are known to positively influence and alter flavonoid metabolism (Liu et al., 2018). Light quality and intensity can affect flavonoid biosynthesis and anthocyanin accumulation, as reported in many fruit crops (Miao et al., 2016; Ma et al., 2019). The spectral wavelengths from the solar radiation are perceived by specific plant photoreceptors that interact upon constitutive photomorphogenesis (COP1) protein, which is one of the key controllers of photomorphogenesis (Wu et al., 2019). COP1 interacts with light-inducible proteins, such as suppressor of phytochrome-A (SPA1), forming complexes that lead to a tightly regulated signal cascade mechanism resulting in changes in the expression of both regulatory and structural genes of the flavonoid biosynthetic pathway (Ma et al., 2021a). The COP1 repressor specifically acts in the degradation of some known positive regulators of anthocyanin biosynthesis, such as elongated hypocotyl 5 (HY5) and MYB transcription factors under altering light and dark conditions (Lu et al., 2017; Ma et al., 2021b). In fruits, it has been shown that MYBs regulate the anthocyanin and flavonol accumulation in response to specific light conditions by mediating light-induced spatiotemporal expression patterns (Zoratti et al., 2014a; Lu et al., 2017).

Supplemental light emitting diodes (LEDs) allow easy modification of light spectrum and are therefore widely used to study light effects and to improve the composition of bioactive compounds in pre- and post-harvest fruits (Kokalj et al., 2019; Panjai et al., 2019). Monochromatic light or combination of light qualities can result in significant variation in the accumulation of specific secondary metabolites in fruits. Specifically, blue and red light at different ratios have been reported to affect biomass, photosynthesis and anthocyanin accumulation in several plant species (Silva et al., 2020; Liang et al., 2021). These two major light wavelengths within PAR range have been extensively studied in many fruit and vegetable crops and shown to promote the secondary metabolites production (Bian et al., 2015).

Many climacteric fruits, such as bananas and tomatoes, are picked before full ripeness to avoid perishability during postharvest storage (Paul et al., 2012). Contrastingly, non-climacteric fruits lack the independent ripening mechanisms, when picked before maturation due to the lack of autocatalytic ethylene biosynthesis. Nevertheless, ripening of non-climacteric fruits is often associated with an increase in another plant hormone, abscisic acid (ABA; Iannetta et al., 2006; Jia et al., 2011; Karppinen et al., 2013; Pilati et al., 2017). In non-climacteric fruits, such as bilberries, previous studies have shown that ABA treatment of unripe detached berries accelerates fruit ripening and anthocyanin accumulation (Chen et al., 2011; Karppinen et al., 2018). The application of sugars, such as sucrose, has also been shown to trigger ripening in detached strawberries, and is often interlinked with endogenous ABA production (Siebeneichler et al., 2020). However, knowledge of the physiological and molecular mechanisms regulating independent ripening without exogenous application of ABA in non-climacteric fruits remain poorly understood.

Among the edible wild berries, bilberry (*Vaccinium myrtillus* L.), which is native to Northern Eurasian boreal regions, is gaining worldwide attention for its rich bioactive and nutritional properties (Colak et al., 2018). Bilberries are widely regarded as one of the richest sources of polyphenolic compounds, especially anthocyanins and other related flavonoids, which accumulate in both skin and flesh during ripening (Jovančević et al., 2011; Korus et al., 2015). In relation to bioactivity, the bilberry polyphenolic compounds are known to possess cardio-protective, anti-carcinogenic, anti-inflammatory properties and antioxidant activities (Seeram, 2008; Pires et al., 2020).

In our recent study, we showed that light quality regulated anthocyanin biosynthesis in naturally ripening bilberries, and that especially red light was able to promote anthocyanin accumulation, mediated through ABA signaling and metabolism (Samkumar et al., 2021). To further deepen our knowledge on the role of light quality on the regulation of flavonoid biosynthesis during non-climacteric bilberry ripening, the effect of light quality was examined in both detached berries and berries ripening naturally on the plant (attached). Thus, this study aimed to add further knowledge toward understanding light responses in fruit ripening that could be useful for designing better adaptation strategies in determining the quality and marketability of fruits and berries.

## Materials and methods

### Plant material and light treatments

Wild bilberry (*V. myrtillus* L.) bushes with intact root systems and forest soil were collected in large boxes (50 cm × 70 cm) during mid-summer (July) when the green berries started to appear from the flowers. The bilberry ecotype used throughout this study was collected from the open vegetation area in Tromsø, Norway (69° 75′N, 19° 01′E). The bilberry bushes were kept in phytotron at 16°C for a few days to acclimatize until the green unripe berries started to increase in size. For the experiments with detached ripening conditions, unripe green berries were picked from the same stand and kept in sterile distilled water until the light treatments. The berries were rinsed a few times with sterile water and 50 berries with 20 ml of sterile water were placed into each Petri dish and sealed with laboratory film (Parafilm M; Sigma-Aldrich, St. Louis, MO, United States).

The bushes in boxes and detached berries in Petri plates were placed inside the chambers covered by photo-reflective sheets with ambient natural light provided from the top. Both experimental setups had three biological replicates per treatment. The temperature was maintained at 16°C inside the chambers. Heliospectra RX30 lamps (Heliopsectra AB., Gothenburg, Sweden) were used to irradiate blue (460 nm), red (660 nm) or far-red (735 nm) light wavelengths inside the chambers. The plants and Petri plates kept under the shaded dark chambers, which received only ambient light from the top, served as a dark control. The plants and Petri plates kept under greenhouse conditions with ample ambient light (400–700 nm) served as white light control for this experiment. The photon fluence rate inside the chambers ranged from 8.0–10.0 μmol m^−2^ s^−1^. The irradiation energy flux (μW cm^−2^) and the distance from light source to plants from all the light treatments were measured using JAZ Spectrometer (Ocean Optics Inc., Orlando, FL, United States). These parameters were used to calculate the relative light intensity and are provided in Supplementary Figure 1.

The changes in berry coloration were assessed and scored visually in detached berries after 2 days (thereafter referred as D2), D5, D7 and D12 from the beginning of light treatments. For RNA isolation, berries (approximately six per replicate) and leaf samples were collected on D0, D2, D4, D7 and D11. For metabolite analyses, fully ripe blue-colored berries were collected; for detached berries, fully ripe berries were collected after 2 weeks, while attached berries on the bushes had fully ripened after 4 weeks from the beginning of the light treatment. A minimum of 20 fully ripe berries per replicate were collected from both experiments for analysis of total anthocyanins and antioxidant assays. After collection, all the berry and leaf samples were immediately frozen in liquid nitrogen and stored at −80°C until used for analyses.

### RNA extraction

The frozen berries and leaves were ground to a fine powder under liquid nitrogen using mortar and pestle. Total RNA was isolated from approximately 120 mg tissue powder by using Spectrum Plant Total RNA kit (Sigma-Aldrich) following the manufacturer’s instructions. The residual DNA was eliminated with on-column digestion using DNase I (Sigma-Aldrich). The RNA was qualified and quantified using a NanoDrop™ 2000c spectrophotometer (Thermo Fischer Scientific, Waltham, MA, United States). First-strand cDNA was synthesized from 1 μg of total RNA using Invitrogen Superscript IV reverse transcriptase (Thermo Fisher Scientific) according to manufacturer’s instructions.

### qRT-PCR analysis

Real-time quantitative reverse transcription PCR (qRT-PCR) analysis was performed with MJ MiniOpticon Real-time PCR system (Bio-Rad, Hercules, CA, United States) using SsoFast™ EvaGreen supermix (Bio-Rad) in 15 μl reaction volume. The PCR conditions were 95°C for 30 s (initial denaturation) followed by 40 cycles at 95°C for 5 s, and 60°C for 10 s. The program was further followed by a melting curve analysis ranging from 65°C to 95°C with an increment of 0.5°C every cycle. All analyses were performed with three biological replicates and two technical replicates. The results were analyzed using CFX connect software (Bio-Rad) using 2^(−ΔΔCt)^ method. The relative expression levels were normalized with housekeeping genes, glyceraldehyde-3-phosphate dehydrogenase (*GAPDH*) or *Actin*. Primer sequences of all the genes used in this study are listed in Supplementary Table 1.

### Analysis of total polyphenols, total anthocyanins, and total flavonoids

Fully ripe berry samples collected from the bushes and Petri plates under light treatments were ground to a fine powder under liquid nitrogen using mortar and pestle and freeze-dried in a lyophilizer (Virtis benchtop-K; SP Scientific, Gardiner, NY, United States). Approximately 100 mg of lyophilized berry powder was subsequently extracted with 1 ml of 100% methanol containing 0.1% (v/v) HCl under constant shaking for 1 h. The samples were centrifuged at 15,000 rpm and the supernatant, with suitable dilution, was further used for the analyses.

Total soluble polyphenols (TPH) were determined according to Doumett et al. (2011) using Folin–Ciocalteu phenol reagent. The absorbance was measured at 740 nm in a spectrophotometer (Smart Spec; Bio-Rad) and the total polyphenol concentrations were calculated from the catechin standard curve. The results were expressed as catechin equivalent in mg g^−1^ dry weight (DW) of berries.

The aluminum chloride-based method was used for the determination of the total flavonoid (TF) content according to Chang et al. (2002). The absorbance was measured at 420 nm in a spectrophotometer (Smart Spec; Bio-Rad). The total flavonoid contents in the samples were calculated from the quercetin standard curve and expressed as quercetin equivalents in mg g^−1^ of DW.

Total monomeric anthocyanins (TMA) were quantified using the pH differential method (Lee et al., 2005). The absorptions from the reaction mixtures in two different pH buffers were measured at 520 and 700 nm. The anthocyanins were calculated based on the differential equation described by Lee et al. (2005). The relative anthocyanin amounts were expressed as cyanidin 3-glucoside equivalents in mg g^−1^ of DW. All the above analyses were performed with three independent biological replicates.

### LC–MS analysis of polyphenols

For liquid chromatography-mass spectrometry (LC–MS) analysis of polyphenols, 100 mg of lyophilized berry powder was used for extractions with 1 ml 100% methanol containing 0.1% (v/v) HCl and the collected supernatants were dried by vacuum evaporation to remove excess methanol. The extracts were resuspended in 500 μl of 20% methanol and filter-sterilized using 0.45 μm filter (Phenomenex, Torrance, CA, United States). The samples were further diluted to 1:10 before injection. The LC–MS system composed of a Dionex Ultimate® 3000 Rapid Separation LC with a micrOTOF QII high-resolution mass spectrometer (Bruker Daltonics, Bremen, Germany). Polyphenols were separated using a Luna Omega C18 (100 × 2.1 mm, 1.6 μm) column maintained at 40°C. The mobile phase consisted of solvents: A = 0.2% formic acid and B = 100% acetonitrile at a flow rate of 400 μl min^−1^. The solvent gradient was: initial composition 95% A, 0–0.5 min; linear gradient to 60% A, 0.5–7 min; linear gradient to 5% A, 7–12 min; composition held at 5% A, 12–16 min; linear gradient to 95% A, 16–16.2 min. The injection volume for the samples and standards was set at 1 μl. The micrOTOF QII parameters were: temperature 225°C; drying N_2_ flow 6 l min^−1^; nebulizer N_2_ 1.5 bar, endplate offset 500 V, mass range 100–1,500 Da, and the data was acquired at 5 scans s^−1^. Negative ion electrospray was used with a capillary voltage of 3,500 V. Polyphenolic concentrations were calculated by comparison to external calibration curves of authentic compounds as standards.

### LC–MS analysis of anthocyanins

Anthocyanins were separated using a Luna Omega Polar C18 (100 × 2.1 mm, 1.6 μm) column maintained at 50°C and the analysis was performed in same LC–MS system as mentioned above. The mobile phase was composed of solvents: A = 5% formic acid in water and B = 100% acetonitrile at a flow rate of 300 μl min^−1^. The solvent gradient was: initial composition 95% A, 0–0.5 min; linear gradient to 85% A, 0.5–10 min; linear gradient to 60% A, 10–20 min; linear gradient to 5% A, 20–25 min; composition held at 95% A, 25–28 min; linear gradient to 95% A, 28–28.2 min; to return to the initial conditions. The injection volume and the micrOTOF QII parameters were same as above. Positive ion electrospray was used with a capillary voltage of 3,000 V. All the anthocyanins were quantified as cyanidin 3-*O*-glucoside (Extrasynthese, Genay, France) equivalents in mg g^−1^ DW.

### FRAP antioxidant activity assay

Ferric reducing antioxidant power (FRAP) assay was used to measure the antioxidant potential in ripening berries according to Benzie and Strain (1996). FRAP reagent was prepared with 10 volumes of 300 mM acetate buffer (pH = 3.6), one volume of 10 mM 2,4,6-tripyridyl-s-triazine in 40 mM HCl and one volume of 20 mM FeCl_3_. Aliquots of 100 μl along with 300 μl of distilled water were added to 3 ml of FRAP reagent pre-warmed at 37°C. After 2 h incubation in the dark, the absorbance was read at 593 nm in a spectrophotometer (Smart Spec; Bio-Rad). The results were calculated from the standard curve prepared from different amounts of FeCl_3_ (100–500 μg) with FRAP reagent and expressed as μmol Fe (II) g^−1^ of DW berries.

### Statistical analysis

Statistically significant differences among gene expression levels between the control and light treatments were determined using Student’s *t*-test in IBM SPSS statistics v26 software package (IBM Corporation, Armonk, NY, United States). The concentrations of anthocyanins and polyphenols between the light treatments and experimental setups were analyzed by one-way analysis of variance (ANOVA) followed by Tukey’s *post-hoc* test. All the visualizations and ANOVA were performed in Origin pro software v2021b (OriginLab Corporation, Northampton, MA, United States).

## Results and discussion

Several studies have shown that specific spectral light wavelengths given as supplemental irradiation during ripening can stimulate the biosynthesis of flavonoids in fruit crops, including species from the *Vaccinium* genus (Arakawa, 1988; Zhou and Singh, 2002; Zoratti et al., 2014b; Zhang et al., 2018a; Samkumar, 2021; Samkumar et al., 2021). However, comprehensive studies investigating the effect of spectral light qualities on non-climacteric fruits, which are ripening either detached or attached to plant, are scarce (Pérez-Llorca et al., 2019). Our results revealed significant differences in the amount and composition of flavonoid compounds between the detached and naturally ripening attached bilberries under different light conditions.

### Red light induces rapid pigment accumulation in berry skin of detached ripened bilberries

**Figure 1:**
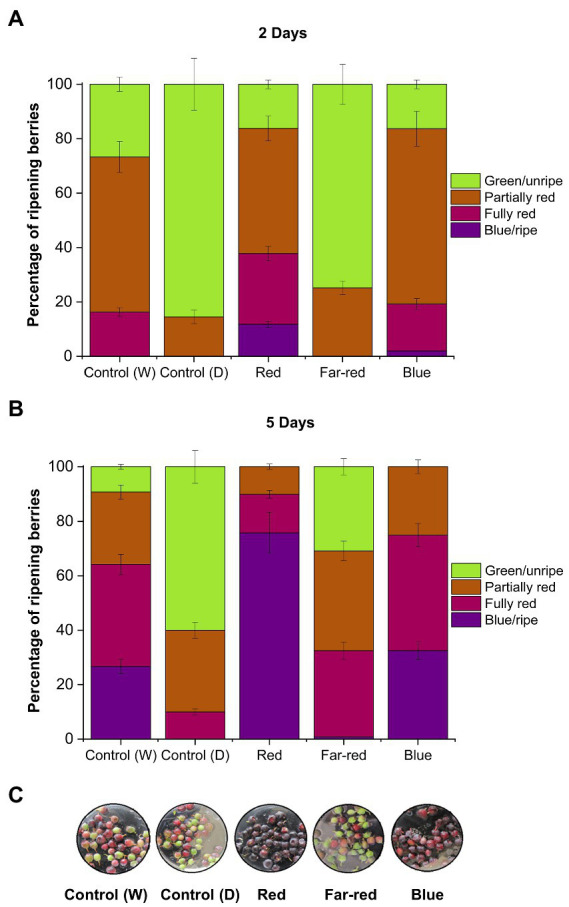
Effect of light spectral quality on berry skin coloration in detached berries after D2 **(A)**, D5 **(B)**, and D7 **(C)** from the beginning of light treatments. Berry coloration is expressed as mean ± SE of three replicates. “Control” represents berries under ambient white light (W) or under dark conditions (D).

The red and blue supplemental light irradiation caused visible changes in berry skin coloration in detached berries, when compared with both controls and far-red light (Figure 1). Red light had the most prominent effect on berry skin coloration. Berries started to turn from green to blue already after D2 under red light (Figure 1A), and most of the berries under red light had turned to fully blue after D5 (Figure 1B). Also, blue light had a positive effect on berry skin coloration to a certain extent and berries reached red/blue coloration after D7 under blue light (Figure 1C). Far-red and dark-treated berries experienced slower coloration during the 7 days light treatment in comparison with other treatments (Figure 1C). All the berries were fully colored after D14 from the beginning of the experiments.

It has been shown earlier in non-climacteric strawberries that berry detachment can accelerate stress-related ripening processes, including rapid coloration (Chen et al., 2014). In some non-climacteric fruit species, the capacity to ripen independently when picked at the green unripe stage has been reported, but fruits were largely deficient in quality attributes, such as aroma, fruit mass and bioactive compounds (Van de Poel et al., 2014). Hence, even if non-climacteric fruits are able to ripen independently of the mother plant when picked before maturation, it is important to determine if the fruit quality and metabolic profile is affected (Adams-Phillips et al., 2004; Cherian et al., 2014). The knowledge concerning independent ripening in non-climacteric fruits corresponding to metabolite accumulation is still limited and differs across species (Cherian et al., 2014).

### Accumulation of phenolic compounds shows differential patterns between detached and attached berries under red and blue light

**Figure 2:**
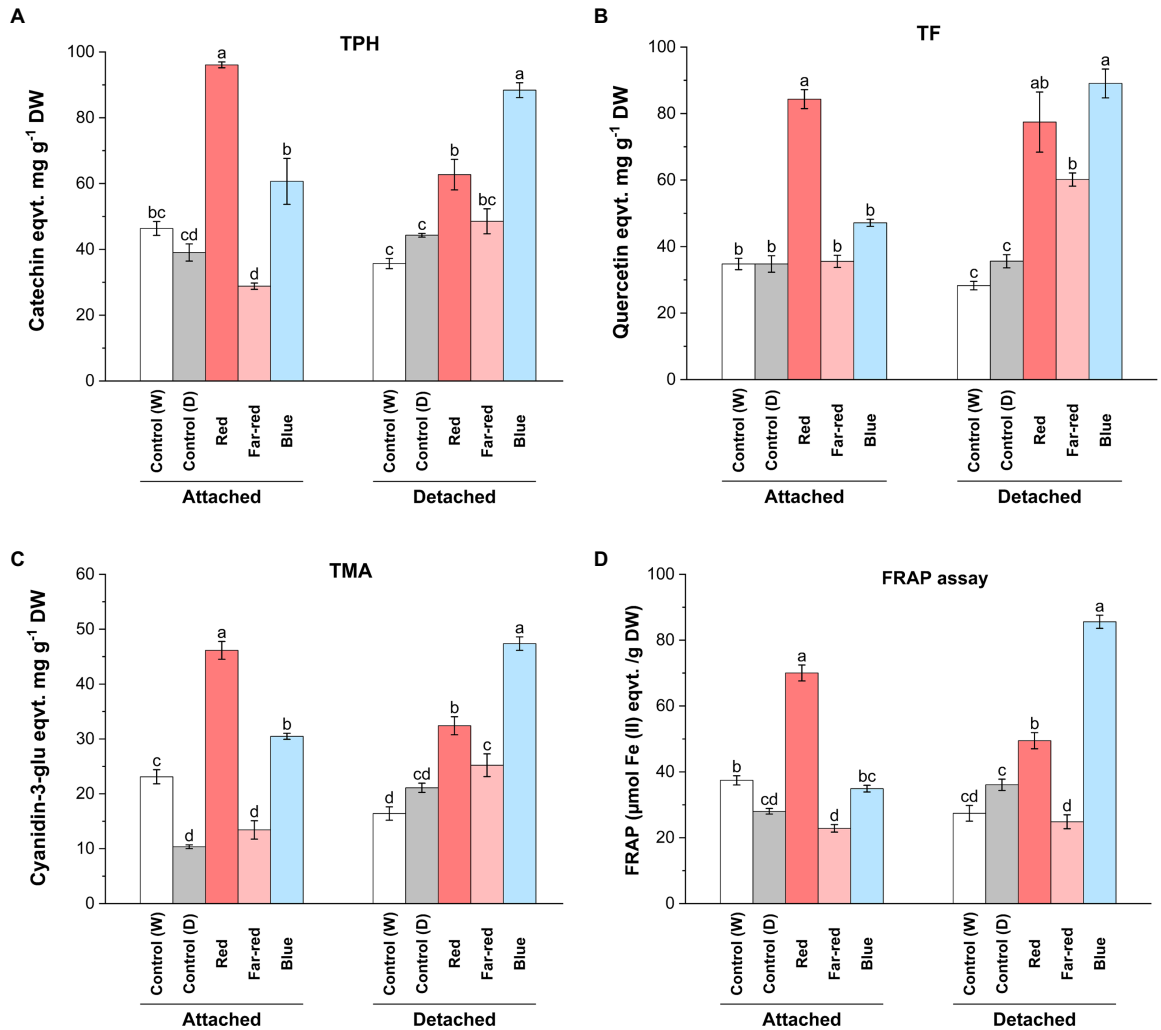
Effect of light spectral quality on total polyphenols (TPH; **A**), total flavonols (TF; **B**), total monomeric anthocyanins (TMA; **C**) and antioxidant activity measured by ferric reducing/antioxidant assay (FRAP; **D**) in detached and attached bilberries. The values represent means ± SE of three biological replicates. Different letters indicate significance between the light treatments using ANOVA with pairwise comparisons followed by Tukey’s *post-hoc* test (*p* ≤ 0.05). DW, dry weight.

From both detached and attached light-treated ripening bilberries, we measured the total soluble polyphenols (TPH), total flavonoids (TF), total monomeric anthocyanins (TMA) and determined the antioxidant potential using the FRAP assay (Figure 2). The results showed the inducing effect of red and blue light on the accumulation of phenolic compounds and the differential effect between detached and attached berries. Both TPH and TF was significantly elevated in attached berries under red light (Figures 2A,B). In detached berries, the highest TPH and TF accumulation was demonstrated under blue light, although red light also induced significant accumulation of phenolic and flavonoid compounds compared to controls (Figures 2A,B). TMA concentration was significantly increased under both red and blue light treatments, with the highest induction in attached berries found under red light, and in detached berries under blue light (Figure 2C). Far-red light could also significantly induce TF and TMA in detached berries but had no effect on TPH (Figures 2A–C).

Both red and blue light treatments have earlier been shown to increase anthocyanin content in many fruit crops under controlled conditions (Lobiuc et al., 2017; Zhang et al., 2018b). Previous studies have shown that red light could selectively induce anthocyanin biosynthesis in fruits, including cranberries (*Vaccinium macrocarpon*; Arakawa, 1988; Zhou and Singh, 2002), whereas blue light has been previously reported in several studies as a major positive influencer of anthocyanin biosynthesis in many other fruit crops. For instance, blue light irradiation promoted the accumulation of bioactive compounds during both pre- and postharvest conditions in tomato and strawberries (Xu et al., 2014; Ngcobo et al., 2020). However, the effect of blue light irradiation specifically to postharvest *Vaccinium* berries, when picked before maturation, have not been reported. When comparing the metabolite analyses with the skin coloration pattern observed in this study, the anthocyanin content was found to be the highest under blue light in detached berries after D14, even if the red light treatment caused a faster response on the coloration of berry skin in detached conditions (Figure 1). This suggests that the anthocyanin profile can be differently regulated in skin and flesh tissues of berries, irrespective of altering light conditions as shown in some earlier studies (He et al., 2010; Guan et al., 2016).

Polyphenolic compounds are known to contribute to the overall antioxidant capacity of berries (Zorzi et al., 2020). The relatively high concentration of phenolic compounds including monomeric anthocyanins and flavonols observed in attached berries under red light and detached berries under blue light contributed to the overall antioxidant potential of the bilberries, as estimated using FRAP assay (Figure 2D). Far-red light had no effect on antioxidant potential of berries (Figure 2D). The role of environmental factors, such as specific light irradiation wavelengths, can potentially determine the total phenolic profile of the berries toward higher antioxidant potential (Ma et al., 2021a). While comparing both ripening conditions upon metabolites accumulation, the biochemical regulatory networks and signals from other source tissues is usually altered in detached conditions, when compared with attached fruits. Some of the major differences could be due to the limitation of photosynthesis, carbon-sugar homeostasis, lack of substrate flow from other tissues such as leaves (source-sink modulation) in detached berries, when compared with attached berries from bushes ripening under natural conditions (Yang et al., 2018). However, the fruit-localized photoreceptors in detached berries might have a prominent role to play in light signal perception and regulating sink strength (Ernesto Bianchetti et al., 2018).

### Delphinidin levels are increased by red light in attached berries and blue light in detached berries

**Figure 3:**
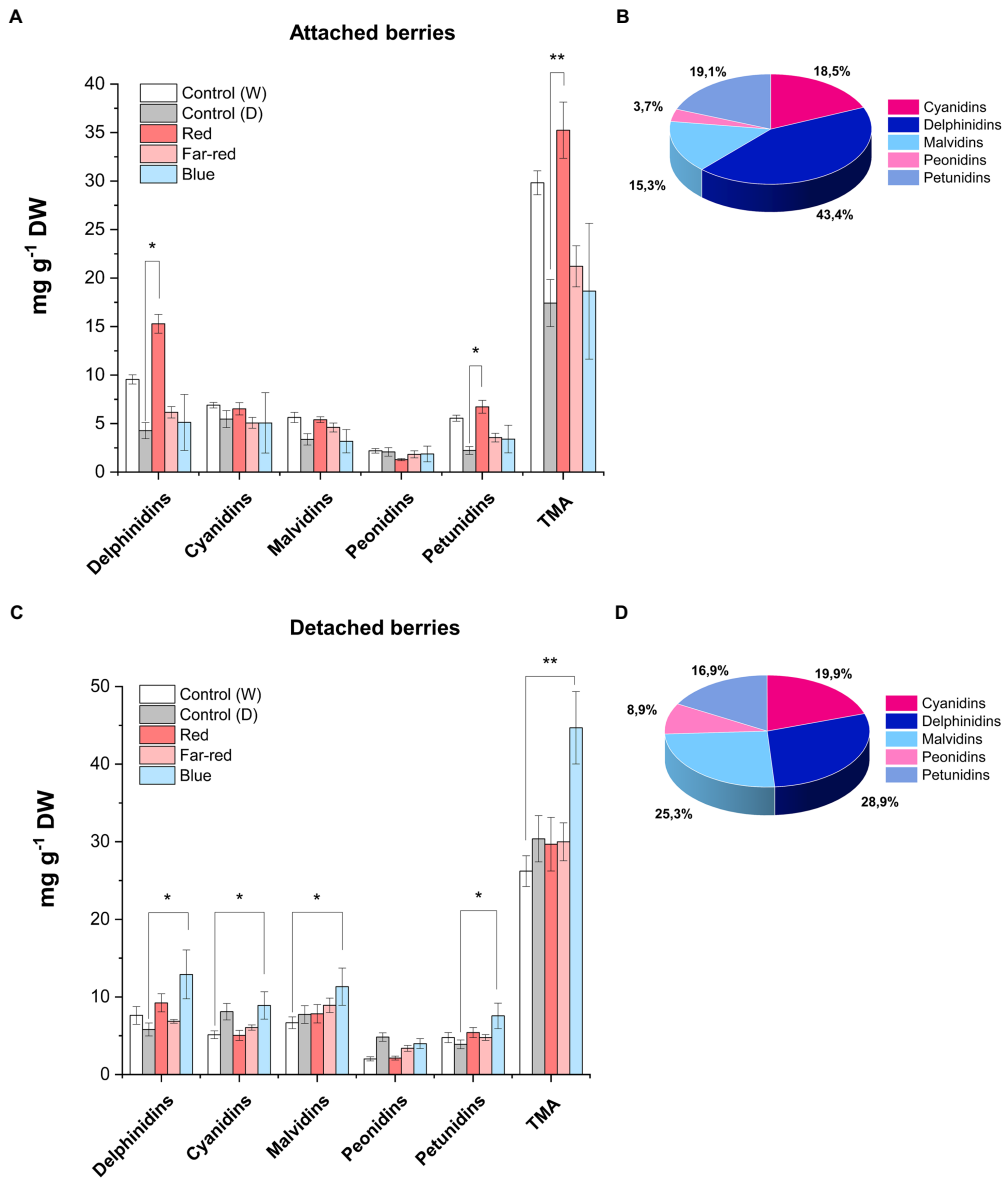
Concentrations of anthocyanins quantified by LC–MS from fully ripe bilberries under different spectral light treatments. Anthocyanin concentration in attached berries under different light conditions **(A)** and the anthocyanin profile in attached berries under red light **(B)**. Anthocyanin concentration in detached berries **(C)** and the anthocyanin profile in detached berries under blue light **(D)**. The concentrations are expressed as an average of three replicates ± SE from glucoside, galactoside and arabinoside-type derivatives from each class of anthocyanin compound. Asterisks indicate significance between the light treatments when measured using ANOVA with pairwise comparisons (*p ≤ 0.05, **p ≤ 0.01). DW, dry weight.

Anthocyanin profiles of bilberries from the different light quality treatments were analyzed by LC–MS. Among the different anthocyanin classes, delphinidin glycosides (glucoside, galactoside and arabinoside) were the most affected by light quality (Figure 3, Supplementary Table 2). The highest concentration of delphinidins in attached berries (15.29 mg g^−1^ DW) was found under the positive influence of red light (Figures 3A,B). In detached berries, the highest concentration of delphinidins (12.90 mg g^−1^ DW) was found under blue light treatment (Figures 3C,D). On the other hand, cyanidins were influenced to a lesser extent by supplemental light treatments in both ripening conditions. In naturally ripening attached berries under red light, the elevated anthocyanin content (35 mg g^−1^ DW) was mostly contributed by the delphinidin class of anthocyanins (Figure 3B). However, the elevated total anthocyanin level detected in detached berries under the blue light (44.40 mg g^−1^ DW) was evenly contributed by delphinidins followed by malvidins, cyanidins, and petunidins, but not peonidins (Figure 3D). It has been previously shown in bilberries that delphinidins are the most reactive anthocyanins toward spectral light treatment (Zoratti et al., 2014b). Our results are in concordant with earlier studies, which have shown that usually the dominant anthocyanin glycosides are the most reactive toward natural or supplemental light. For example, delphinidins in rabbiteye blueberries and pelargonidins in strawberries were shown to be the most reactive to light (Zhang et al., 2018a; Guo et al., 2022).

Our findings are in agreement with a previous study in non-climacteric strawberries, where the anthocyanin levels were found to be higher in postharvest fruits than naturally ripening attached berries (Siebeneichler et al., 2020). The authors suggested a dual role for ABA hormone, by acting as an elevated stress marker on detached conditions, but actively involved in ripening process when fruits are attached to plants (Vighi et al., 2019; Siebeneichler et al., 2020). Thus, classifying the nature of fruit ripening based on hormonal release or response to exogenous application at onset of ripening is contentious, since it widely varies across fruit species (Trainotti et al., 2005; Symons et al., 2012; Luo et al., 2014). For instance, another study in postharvest strawberry showed that certain ripening attributes mimicked climacteric fruit characteristics *in planta* (Iannetta et al., 2006).

### Red and far-red light positively influence accumulation of flavonols in both detached and attached berries

**Figure 4:**
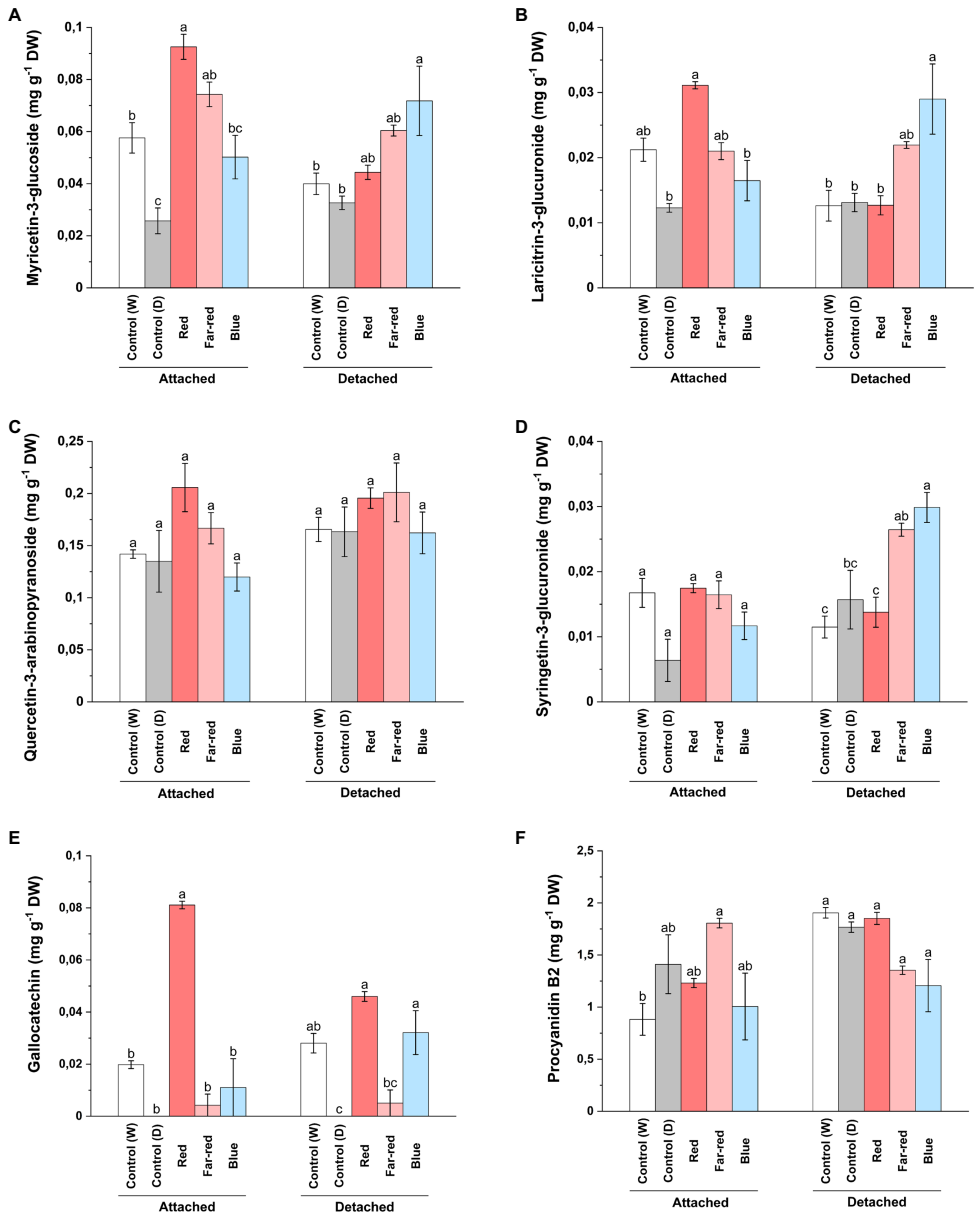
Concentrations of myricetin 3-glucoside **(A)**, laricitrin 3-glucuronide **(B)**, quercetin-3-arabinopyranoside **(C)**, syringetin 3-glucuronide **(D)**, gallocatechin **(E)**, and procyanidin B2 dimer **(F)** under different light treatments quantified using LC–MS. The concentrations are expressed in average of three biological replicates ±SE. Different letters indicate significance between the light treatments using ANOVA with pairwise comparisons followed by Tukey’s *post-hoc* test (*p* ≤ 0.05). DW, dry weight.

In contrast to our findings on anthocyanin compounds, red light influenced accumulation of total flavonols in both ripening conditions (Supplementary Table 3). However, some delphinidin branch-derived flavonols were specifically influenced by red light treatment in attached berries (Figure 4) and was consistent with our anthocyanin analysis. On the other hand, blue light was also found to increase major flavonol compounds in detached conditions (Supplementary Table 3). It was closely followed by far-red light, which also influenced some flavonols such as syringetin glycosides (Figure 4). LC–MS analysis showed that myricetin 3-glucoside and its derivate, laricitin 3-glucuronide, derived from delphinidin branch pathway was significantly increased in attached berries under red light treatment compared to other light treatments (Figures 4A,B). On detached conditions, both blue and far-red light treatment had a positive influence on most of the phenolic compounds (Supplementary Table 3). Our results are in accordance with a study, which showed that quercetin compounds can be directly influenced by continuous far-red light and accumulation of quercetin compounds is regulated separately from anthocyanin accumulation through phytochrome-induced biosynthesis (Beggs et al., 1987). In our study, quercetin 3-arabinopyranoside did not show any significant increase under the light treatments compared to controls (Figure 4C). Syringetin 3-glucuronide was found to be significantly increased in detached berries by blue light followed by far-red light treatment (Figure 4D). Overall, the total flavonol concentration was found to be higher in red light treatment for attached berries (2.40 mg g^−1^ DW) compared with any other light treatments (Supplementary Table 3). In detached berries, red light (2.54 mg g^−1^ DW), followed by far-red light (2.46 mg g^−1^ DW), increased the total flavonols concentration. Our results are in accordance with a previous study on bilberry, which showed the total flavonol concentrations corresponding to the effect of monochromatic red and far-red light in ripe bilberries (Zoratti et al., 2014b).

Concerning proanthocyanidins, gallocatechin was detected in very low levels in both light treatments and controls, but increased almost fourfold under red light treatment in attached berries (Figure 4E). The procyanidin dimers (B1, B2 and C1) were not found to be significantly affected by the light treatments (Figure 4F; Supplementary Table 3). Procyanidin C1 and B2 concentrations were slightly increased in attached berries under the influence of far-red light. In a recent study on strawberry genotypes, it was shown that enhanced red light affected both anthocyanin and proanthocyanidin biosynthesis more than blue light, but the accumulation pattern differed during fruit development based on anthocyanidin reductase (*ANR*) expression (Zhang et al., 2018b).

The effect of far-red light was also seen on caffeoyl-4-glucoside concentration on both detached and attached berries (Supplementary Table 2). Nandinaside-A (C_22_H_22_O_10_), another caffeoyl-glucoside, was detected in increased concentration only in dark shaded control samples (Supplementary Table 3). Leucocyanidin showed a similar trend compared to the total anthocyanin amounts; red light influencing concentration in attached berries and blue light in detached berries (Supplementary Table 2). Epicatechin and trans-p-coumaric acid were found to be increased under far-red light, whereas catechin concentrations were elevated by red light in naturally ripening berries (Supplementary Table 3).

### Flavonoid biosynthetic genes are differentially regulated by the key MYB genes in attached and detached berries

**Figure 5:**
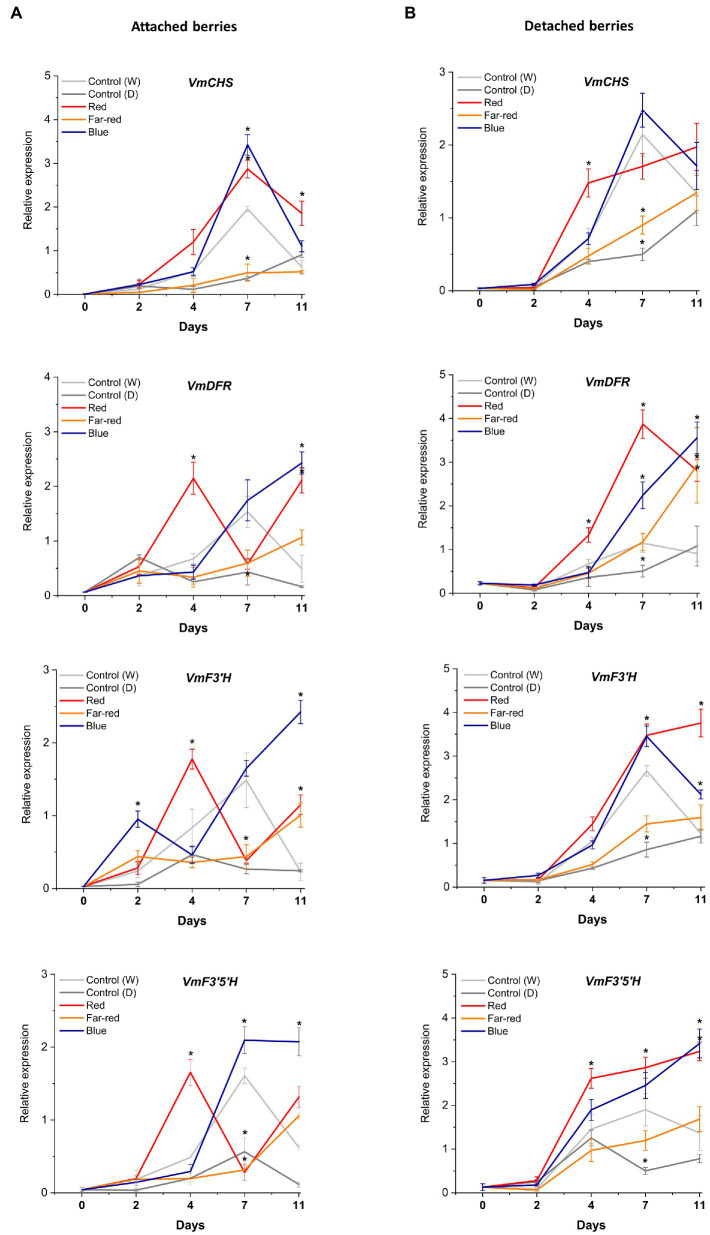
Effect of light spectral treatments on gene expression of early flavonoid biosynthetic genes chalcone synthase (*VmCHS*), dihydroflavonol 4-reductase (*VmDFR*), flavonoid 3*′* hydroxylase (*VmF3’H*), flavonoid 3*′*5*’* hydroxylase (*VmF3’5’H*) in attached berries **(A)** and in detached berries **(B)**. The expression levels were normalized to housekeeping gene glyceraldehyde 3-phosphate dehydrogenase (*VmGAPDH*) or *actin*. Expressed in average of three biological replicates ±SE and significant differences between control and light treatments were analyzed by comparison of means using Student’s *t*-test (in asterisks*) with value of *p* ≤ 0.05.

**Figure 6:**
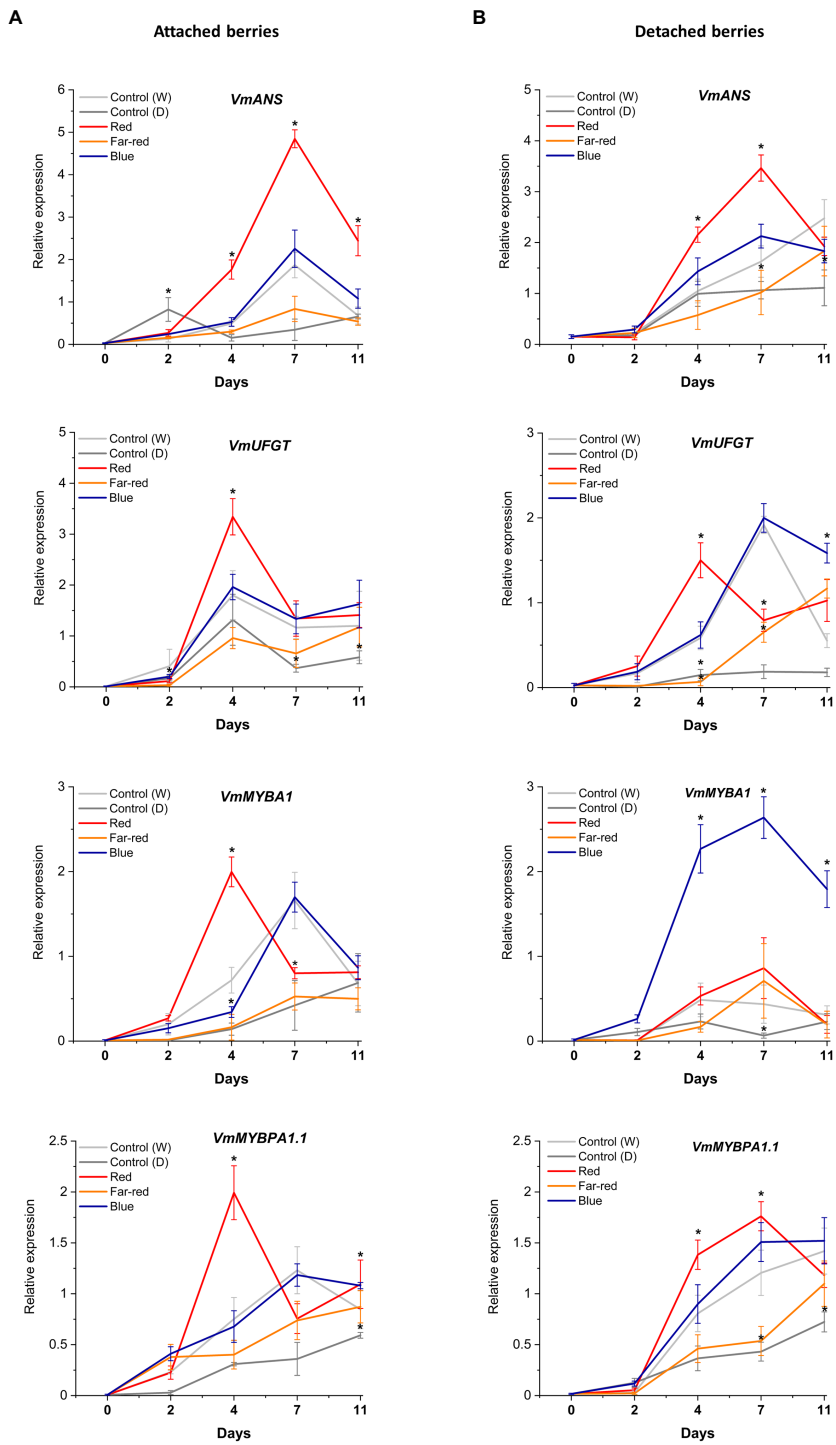
Effect of light spectral treatments on gene expression of late flavonoid biosynthetic genes (*VmANS* and *VmUFGT*) and key regulatory genes (*VmMYBA1*, *VmMYBPA1.1*) in attached berries **(A)** and in detached berries **(B)**. The expression levels were normalized to the housekeeping gene *VmGAPDH* or *actin*. Expressed in average of three biological replicates ±SE and significant differences between control and light treatments were analyzed by comparison of means using Student’s *t*-test (indicated in asterisks*) with value of *p* ≤ 0.05.

The relative expression of key flavonoid biosynthetic genes in both attached and detached berries was analyzed over the time-course of the light experiments (Figures 5, 6). Both red and blue light increased the expression of all the studied structural genes at the beginning of the experiment in both detached and attached berries from D2 onwards. Interestingly, far-red light also increased the expression of many biosynthetic genes, especially on detached berries. It should be noted that two enzymes, *DFR* and *FLS*, compete for dihyfroflavonols as substrate in flavonoid pathway, which direct biosynthesis either toward anthocyanins and proanthocyanidins or flavonols (Davies et al., 2003). This is in agreement with our results that far-red could also positively influence accumulation of some of the major flavonols, such as quercetin, myricetin and syringetin glycosides, in detached berries (Figure 4). The expression of cyanidin and delphinidin branchpoint genes (*VmF3'H* and *VmF3'5'H*) were found at significantly higher level at D4 under red light in attached berries compared to detached berries, indicating substrate diversion to anthocyanin accumulation together with *VmDFR* and *VmUFGT*, which also showed very similar expression pattern (Figure 5A). It can be concluded that red light induced faster and shorter response, but blue light appeared to increase the expression of early biosynthetic genes at later time points in attached berries.

In detached berries, *VmF3'5'H* and *VmDFR* expression steadily increased after D4 under both red and blue light (Figure 5B), and were thus directly correlated with the increased anthocyanin accumulation shown in Figure 3. The positive influence of red light on *VmF3'H* and *VmDFR* gene expression levels could also be associated with the accumulation of flavonols in detached berries as shown above. The expression of the late biosynthetic genes, such as *VmANS*, showed similar trends in both experimental setups (Figures 6A,B). *VmUFGT* transcript abundance, on the other hand, increased under red light in attached berries after D4, and under blue light in detached berries after D7 of irradiation (Figures 6A,B).

Interestingly, one of the key anthocyanin regulatory transcription factors, *VmMYBA1*, was found to be expressed in fivefold higher in detached berries under blue light compared to controls (Figures 6A,B) and correlating with *VmUFGT* expression. Likewise, under red light in attached berries, both *VmMYBA1* and *VmMYBPA1.1* had the highest expression levels after D4 of treatment (Figures 6A,B), similar to *VmANS* and *VmUFGT* expression. An earlier study also showed that light quality could differentially regulate anthocyanin accumulation by affecting the downstream late biosynthetic genes, such as *DFR* and *UFGT* (Yang et al., 2018). This suggests that certain key biosynthetic and regulatory genes can perform a light-dependent regulation of flavonoid biosynthesis in a different manner under different ripening conditions (Steyn et al., 2004). It also indicates that *MYBA1* might be the key factor driving the blue light-mediated anthocyanin accumulation in detached and independently ripening berries by specifically interacting with the late biosynthetic genes. *MYBA1* has been earlier been shown as one of the key regulators of anthocyanin biosynthesis in *Vaccinium* berries, activating the promoters of *DFR* and *UFGT* (Plunkett et al., 2018; Die et al., 2020). Both *VmMYBA1* and *VmMYBPA1.1* showed very similar expression patterns in naturally ripening berries under the influence of red light, suggesting that both of these MYB transcription factors might co-regulate anthocyanin biosynthesis toward the delphinidin branch (Figures 6A,B). It has been recently reported that these two MYBs were the key regulators of anthocyanin biosynthesis during bilberry ripening (Karppinen et al., 2021; Lafferty et al., 2022).

We did not observe significant influence to expression patterns of most of the flavonoid biosynthesis related genes in bilberry leaves by light treatments. Only *VmCHS* expression increased under red light treatment in leaves (Supplementary Figure 2). It appeared to have a synergistic effect to that of expression found in berries, with the highest level reached under blue light after D2 and red light increasing the expression rapidly from D4 onwards (Supplementary Figure 2A). *VmMYBPA1.1* also showed a significantly higher expression level early in response to blue light treatment (Supplementary Figure 2B), but under red light the expression steadily increased toward higher levels after D7. The leaves are mostly regarded as by-product in horticultural crops, but in some species such as blueberry, the polyphenolic constituents in leaves are often found to be higher than fruits (Li et al., 2013; Zhu et al., 2013). In terms of molecular regulation and signaling, the most important role of leaves in attached ripening conditions could be modulating the light signaling response cascades, alongside maintaining the source-sink balance from photosynthesis, which could potentially affect berry coloration and anthocyanin composition (Mota et al., 2010; Pastore et al., 2011; Bobeica et al., 2015).

### Red light responses in attached berries are indicated to be mediated through COP1

**Figure 7:**
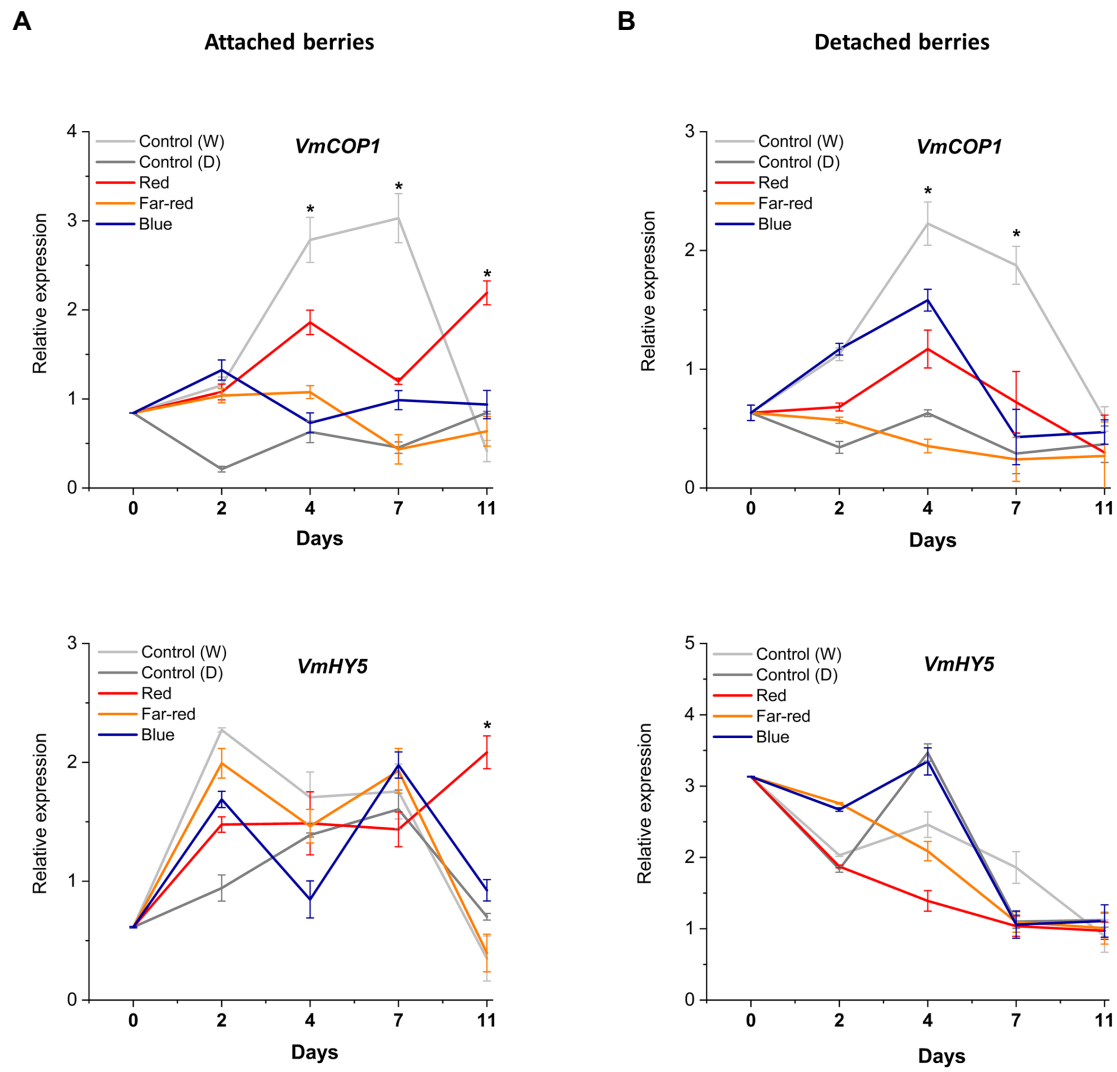
Effect of light spectral treatments on the expression of photomorphogenesis related genes (*VmCOP1* and *VmHY5*) in attached berries **(A)** and in detached berries **(B)**. The expression levels were normalized to the housekeeping gene *VmGAPDH* or *actin*. Expressed in average of three biological replicates ±SE and significant differences between control and light treatments were analyzed by comparison of means using Student’s *t*-test (indicated in asterisks*) with value of *p* ≤ 0.05.

The effect of spectral light quality on the COP1-related regulatory mechanisms was not clearly influenced in detached ripening berries, including *HY5* expression. The bilberry *COP1* gene, alongside *VmHY5*, showed little response to tested light treatments (Figures 7A,B). On the contrary, in attached berries under red light, photomorphogenesis was likely mediated *via VmCOP1*, the key signaling component and repressor of anthocyanin biosynthesis (Wu et al., 2019; Ma et al., 2021b). Generally, in high light conditions, COP1 is exported to cytosol, that allows positive regulators, such as HY5, to accumulate in the nucleus (Zoratti et al., 2014a). In our experiments, its expression levels tended to increase after D7 under red light treatment (Figure 7B). The key interaction between COP1 and HY5 has been shown to determine the inhibitory effects and hyperaccumulation of anthocyanins under low and high light (Maier and Hoecker, 2015). Hence, detached berries ripening independently compared with berries attached to the plant might have different regulatory mechanisms and signaling routes to protect the tissues from strong monochromatic light environments during ripening. The former ripening condition showed a very strong systemic response by producing high levels of photoprotective anthocyanin compounds, which also reflected in its overall antioxidant capacity.

The most interesting finding in our study was how both the light qualities were differentially perceived and regulated anthocyanin accumulation in detached and non-detached berries. For instance, the elevated total anthocyanin level mediated by blue light in detached ripening berries, which was even higher than in berries attached to the bush (Figures 3A,B; Supplementary Table 2). This was found peculiar and interesting, given the nature of non-climacteric fruit ripening, also considering that detached berries lacked autonomous hormonal signaling and were totally devoid of substrate flow from source tissues.

In addition to the differential responses that we have observed from the light spectral quality, further studies that compare fruit ripening, with or without signals from the mother plant, will bridge the gap in understanding independent ripening mechanisms and subsequent accumulation of phytochemicals or bioactive compounds. Our results strongly suggest that detached ripening in bilberries is quite distinct from *in vivo* natural ripening under specific spectral light quality.

## Conclusion

In this study, we have shown that the response to simulated light quality treatments is different in naturally ripening attached and in detached berries. Our results clearly showed that flavonoid biosynthesis during ripening was positively influenced but differentially regulated in controlled experimental conditions. Blue light induced the highest anthocyanin accumulation in detached berries, while red light stimulated anthocyanin biosynthesis the most in naturally ripening attached berries. The overall anthocyanin content was found to be the highest and significantly elevated in blue light-treated detached berries. The composition of anthocyanins was evenly distributed among the major anthocyanin classes in detached conditions, unlike the accumulation of most reactive delphinidin branch and its derived compounds myricetin and laricitrin glycosides in attached berries. The current study has shown that *Vaccinium* berries could be used for further investigation of molecular mechanisms and signaling between the mother plant and naturally ripening fruit. Light treatments with both supplemental blue and red wavelengths could be also considered in future cultivation practices for improved anthocyanin content in blue-colored berries.

## Data availability statement

The original contributions presented in the study are included in the article/Supplementary material, and further inquiries can be directed to the corresponding author.

## Author contributions

LJ, KK, and AS conceptualized the project. AS performed the experiments, designed methodologies, analyzed the data, and wrote the manuscript draft. TM performed the anthocyanin and polyphenolic profiling with LC–MS. LJ, KK, IM, and RE contributed in editing and proofreading of the manuscript draft. All authors contributed to the article and approved the submitted version.

## Funding

The research visit was supported by the New Zealand Ministry for Business, Innovation, and Employment (MBIE) Endeavour programme “Filling the Void: boosting the nutritional content of NZ fruit” (contract no. C11X1704). The work was also financially supported by NordPlant (NordForsk grant no. 84597).

## Conflict of interest

Authors TM and RE are employed by The New Zealand Institute for Plant & Food Research Ltd.

The remaining authors declare that the research was conducted in the absence of any commercial or financial relationships that could be construed as a potential conflict of interest.

## Publisher’s note

All claims expressed in this article are solely those of the authors and do not necessarily represent those of their affiliated organizations, or those of the publisher, the editors and the reviewers. Any product that may be evaluated in this article, or claim that may be made by its manufacturer, is not guaranteed or endorsed by the publisher.
