# Supplementary material for: Flavonoid biosynthesis is differentially altered in detached and attached ripening bilberries in response to spectral light quality
Source: Front Plant Sci. 2022 Jul 22;13:969934. doi: 10.3389/fpls.2022.969934 (PMC9355381; doi:10.3389/fpls.2022.969934)
Supplement: Supplementary file 1 [file Table_1.DOCX]

**Supplementary Information**

**Flavonoid biosynthesis is differentially altered in detached and attached ripening bilberries in response to spectral light quality**

**Amos Samkumar ^a,^ *, Katja Karppinen ^a^, Tony K. McGhie ^b^, Richard V. Espley ^c^, Inger Martinussen ^d^, Laura Jaakola ^a, d^**

^a^ Department of Arctic and Marine Biology, UiT The Arctic University of Norway, Tromsø, Norway

^b^ The New Zealand Institute for Plant & Food Research Ltd., Palmerston North, New Zealand

^c^ The New Zealand Institute for Plant & Food Research Ltd., Auckland, New Zealand

^d^ Department of Horticulture, Norwegian Institute of Bioeconomy Research, Ås, Norway

*Corresponding author. *E-mail address*: [amos.s.premkumar@uit.no](mailto:amos.s.premkumar@uit.no) (A. Samkumar)

**Number of figures: 2**

**Number of tables: 4**


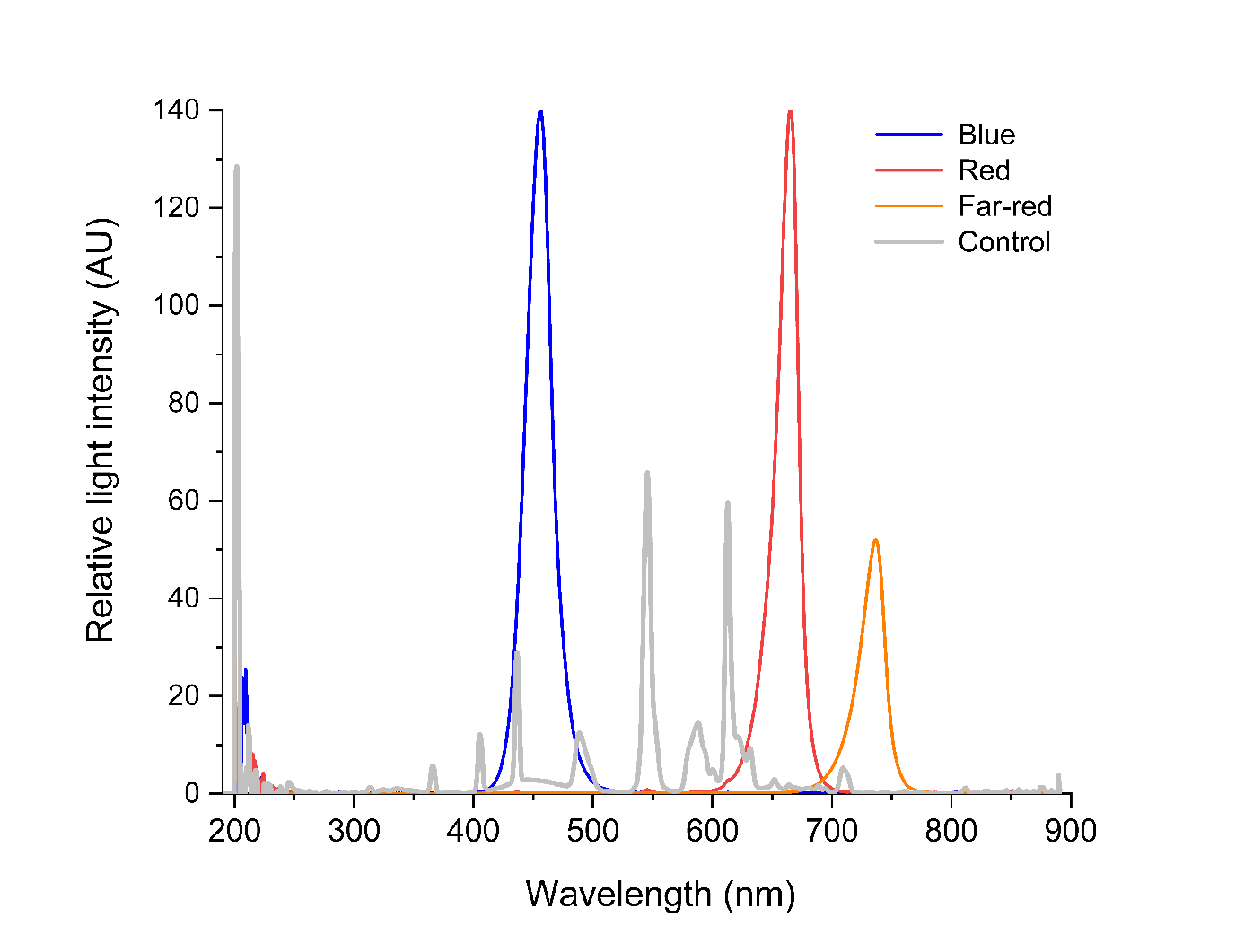


**Figure 1.** Supplemental blue (460 nm), red (660 nm) and far-red (735nm) light treatments provided for bilberry bushes and detached berries with Heliospectra LED lamps alongside control (400-700 nm). Relative intensities of light spectra from the treatments were expressed as arbitrary units (AU).


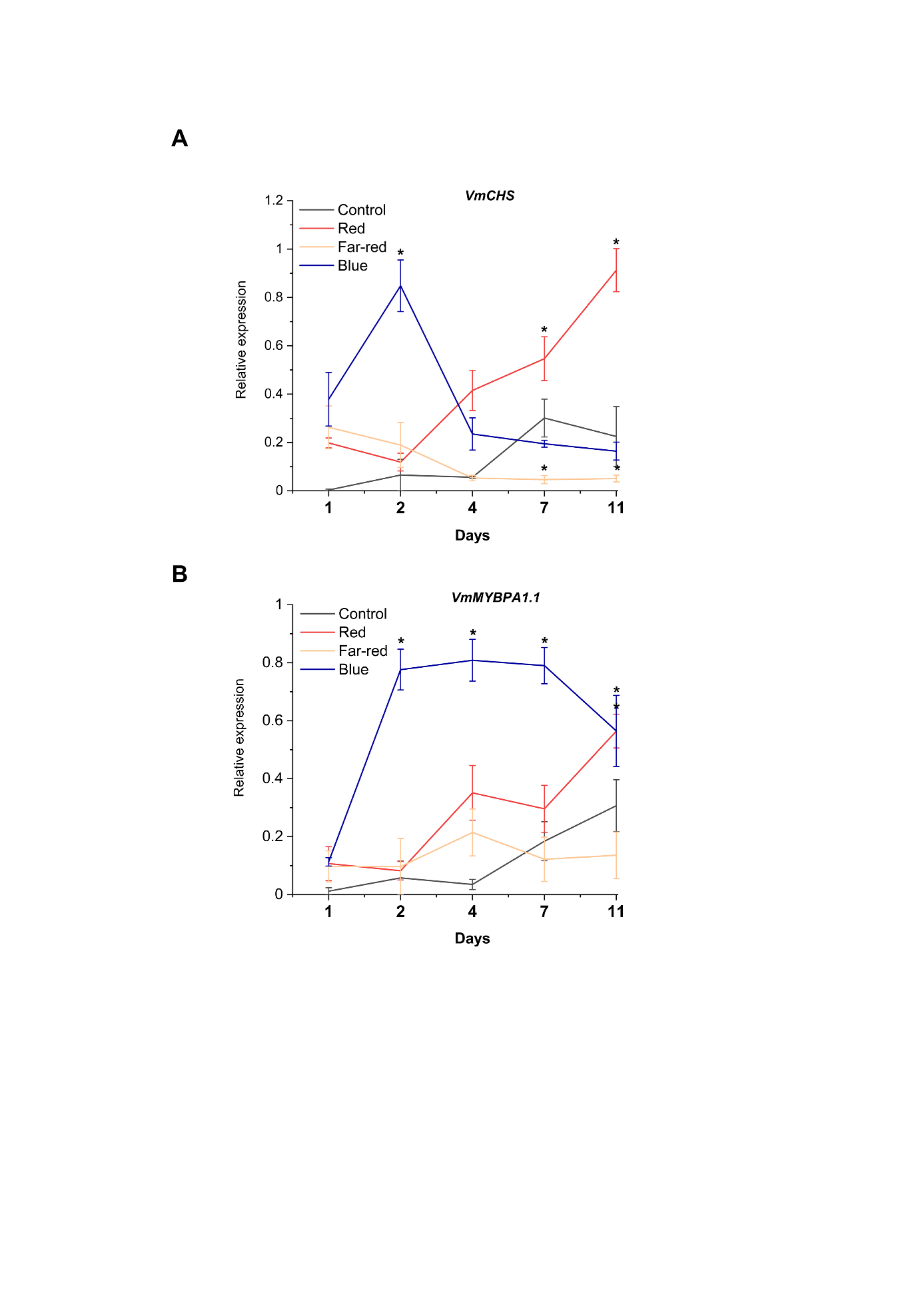


**Figure 2.** Effect of light spectral treatment on gene expression of a major flavonoid biosynthetic gene and a regulatory gene in bilberry leaves, Chalcone synthase (*VmCHS*) **(A)**, R2R3-MYB transcription factor (*VmMYBPA1.1*) **(B)**. The expression levels are normalized to the reference gene *VmGAPDH* (Glyceraldehyde 3-phosphate dehydrogenase). Error bars represents ±SE of three biological replicates and significant differences between control and light treatments were analyzed by comparison of means using student’s t-test (indicated in asterisks*) with p-value ≤ 0.05.

**Supplementary Table 1.** List of primers used in qRT-PCR analysis.

| **Gene** | **Forward primer sequence** | **Reverse primer sequence** |
| --- | --- | --- |
| *VmCHS* | CCAAGGCCATCAAGGAATG | TGATACATCATGAGTCGCTTCAC |
| *VmF3'H* | TTCTTCGACACCCGAAAGTC | TCGAACCCTTTGGAATGAAG |
| *VmF3'5'H* | GATTGCGTGGATGGACTTACA | AAATCTGGGTTCCCTTTACGC |
| *VmDFR* | GAAGTGATCAAGCCGACGAT | ATCCAAGTCGCTCCAGTTGT |
| *VmANS* | GCAACTCTTCTACGAGGGCAAA | CCTGTGGAGAATGCTCTTGCAC |
| *VmUFGT* | CATCCAAACCCTGTTCCCATCC | TCATCCCTGCCTTCAAGCTCTC |
| *VmMYBA1* | CTCGACCACAAACCTTGTCCA | GCCTCCTCATTTGATCCGTCA |
| *VmMYBPA1.1* | GGACATTCAACGCCAATCTGGT | CGGCAAAGGAATCCAACTGAAG |
| *VmCOP1* | TGAGAAATGTCAGCCAACCA | CTCTAAATGTGCGCAGTGGA |
| *VmHY5* | GGGAGGAAGTAAGGTCCAAATG | TATAGGGTTACCGGGAGGAATG |
| *VmGAPDH* | CAAACTGTCTTGCCCCACTT | CAGGCAACACCTTACCAACA |
| *VmActin* | TTCCCTGGGATTGCTGATAG | GGTCTTGGCAATCCACATCT |

**Supplementary Table 2**. Concentration of different classes of anthocyanin compounds (mg g^-1^ DW) quantified by LC-MS in fully ripe bilberries at the end of spectral light treatment from both attached and detached berries.

|  | **Attached berries**  **Control (W) Control (D) Far-Red Red Blue** | **Detached berries**    **Control (W) Control (D) Far-Red Red Blue** |
| --- | --- | --- |
| **DEL_GLU**  **DEL_GAL**  **DEL_ARA**  Total_DEL | \| \| 2.7±0.15^a^ \| 1.5±0.31^a^ \| 2.0±0.15^a^ \| 3.8±0.06^a^ \| 2.2±0.71^a^ \| \| --- \| --- \| --- \| --- \| --- \| \| 2.7±0.13^ab^ \| 1.2±0.27^b^ \| 1.9±0.16^b^ \| 5.4±0.23^a^ \| 1.4±1.03^b^ \| \| 4.0±0.18^ab^ \| 1.5±0.23^b^ \| 2.1±0.26^b^ \| 6.0±0.66^a^ \| 1.3±1.14^b^ \| \| **9.55** \| **4.28** \| **6.16** \| **15.29** \| **5.12** \| \| \| --- \| --- \| --- \| --- \| --- \| --- \| --- \| --- \| --- \| --- \| --- \| --- \| --- \| --- \| --- \| --- \| --- \| --- \| --- \| --- \| --- \| | \| \| 1.7±0.32^a^ \| 1.7±0.20^a^ \| 1.7±0.10^a^ \| 2.1±0.37^a^ \| 3.9±1.30^a^ \| \| --- \| --- \| --- \| --- \| --- \| \| 1.6±0.24^a^ \| 1.5±0.17^a^ \| 1.6±0.09^a^ \| 2.0±0.33^a^ \| 3.8±1.22^a^ \| \| 4.1±0.56^a^ \| 2.5±0.46^a^ \| 3.5±0.03^a^ \| 5.0±0.46^a^ \| 5.0±0.61^a^ \| \| **7.63** \| **5.81** \| **6.87** \| **9.24** \| **12.90** \| \| \| --- \| --- \| --- \| --- \| --- \| --- \| --- \| --- \| --- \| --- \| --- \| --- \| --- \| --- \| --- \| --- \| --- \| --- \| --- \| --- \| --- \| |
| **CYA_GLU**  **CYA_GAL**  **CYA_ARA**  Total_CYA  **MAL-GLU**  **MAL-GAL**  **MAL-ARA**  Total_MAL | \| 2.4±0.12^a^ \| 1.9±0.30^a^ \| 1.9±0.26^a^ \| 1.7±0.19^a^ \| 2.4±0.81^a^ \| \| --- \| --- \| --- \| --- \| --- \| \| 2.3±0.10^a^ \| 1.4±0.27^a^ \| 1.5±0.21^a^ \| 2.3±0.30^a^ \| 1.4±1.33^a^ \| \| 2.0±0.04^a^ \| 2.0±0.31^a^ \| 1.5±0.07^a^ \| 2.4±0.14^a^ \| 1.1±0.95^a^ \| \| **6.90** \| **5.46** \| **5.07** \| **6.53** \| **5.07** \| \| 3.2±0.25^a^ \| 2.1±0.40^a^ \| 2.7±0.14^a^ \| 2.7±0.13^a^ \| 2.0±0.66^a^ \| \| 1.1±0.14^a^ \| 0.47±0.10^a^ \| 0.79±0.10^a^ \| 1.2±0.70^a^ \| 0.6±0.31^a^ \| \| 1.2±0.12^a^ \| 0.69±0.07^a^ \| 1.0±0.21^a^ \| 1.3±0.09^a^ \| 0.5±0.22^a^ \| \| **5.63** \| **3.37** \| **4.60** \| **5.40** \| **3.18** \| | \| 1.5±0.16^a^ \| 2.9±0.31^a^ \| 1.9±0.15^a^ \| 1.3±0.16^a^ \| 2.9±0.78^a^ \| \| --- \| --- \| --- \| --- \| --- \| \| 1.2±0.07^a^ \| 2.2±0.24^a^ \| 1.6±0.09^a^ \| 1.1±0.11^a^ \| 2.6±0.68^a^ \| \| 2.3±0.26^a^ \| 2.8±0.50.^a^ \| 2.4±0.08^a^ \| 2.5±0.37^a^ \| 3.3±0.29^a^ \| \| **5.13** \| **8.10** \| **6.05** \| **5.05** \| **8.91** \| \| 2.7±0.37^a^ \| 3.9±0.43^a^ \| 3.6±0.48^a^ \| 3.2±0.53^a^ \| 5.4±1.42^a^ \| \| 0.9±0.09^b^ \| 1.2±0.16^ab^ \| 1.4±0.20^ab^ \| 1.1±0.15^b^ \| 2.2±0.49^a^ \| \| 2.9±0.28^a^ \| 2.5±0.53^a^ \| 3.8±0.23^a^ \| 3.4±0.49^a^ \| 3.5±0.46^a^ \| \| **6.68** \| **7.74** \| **8.92** \| **7.84** \| **11.32** \| |
| **PEO-GLU**  **PEO-GAL**  **PEO-ARA**  Total_PEO  **PET-GLU**  **PET-GAL**  **PET-ARA**  Total_PET | \| \| 1.5±0.15^a^ \| 1.4±0.32^a^ \| 1.3±0.25^a^ \| 0.7±0.07^a^ \| 1.4±0.51^a^ \| \| --- \| --- \| --- \| --- \| --- \| \| 0.3±0.04^a^ \| 0.2±0.07^a^ \| 0.2±0.05^a^ \| 0.2±0.01^a^ \| 0.2±0.18^a^ \| \| 0.2±0.03^a^ \| 0.3±0.02^a^ \| 0.2±0.05^a^ \| 0.2±0.02^a^ \| 0.1±0.10^a^ \| \| **2.19** \| **2.08** \| **1.83** \| **1.29** \| **1.87** \| \| 2.8±0.16^a^ \| 1.2±0.25^a^ \| 1.9±0.23^a^ \| 2.8±0.27^a^ \| 2.1±0.64^a^ \| \| 1.2±0.05^ab^ \| 0.4±0.08^b^ \| 0.7±0.09^ab^ \| 1.7±0.15^a^ \| 0.6±0.39^b^ \| \| 1.5±0.08^ab^ \| 0.5±0.05^b^ \| 0.8±0.11^b^ \| 2.1±0.23^a^ \| 0.6±0.39^b^ \| \| **5.55** \| **2.23** \| **3.55** \| **6.73** \| **3.41** \| \| \| --- \| --- \| --- \| --- \| --- \| --- \| --- \| --- \| --- \| --- \| --- \| --- \| --- \| --- \| --- \| --- \| --- \| --- \| --- \| --- \| --- \| --- \| --- \| --- \| --- \| --- \| --- \| --- \| --- \| --- \| --- \| --- \| --- \| --- \| --- \| --- \| --- \| --- \| --- \| --- \| --- \| | \| 1.2±0.17^b^ \| 3.0±0.31^a^ \| 1.9±0.24^ab^ \| 1.2±0.15^b^ \| 2.5±0.40^ab^ \| \| --- \| --- \| --- \| --- \| --- \| \| 0.2±0.02^b^ \| 0.6±0.10^a^ \| 0.4±0.06^ab^ \| 0.2±0.03^b^ \| 0.6±0.10^ab^ \| \| 0.5±0.06^b^ \| 1.0±0.13^a^ \| 0.9±0.06^ab^ \| 0.6±0.06^b^ \| 0.8±0.12^ab^ \| \| **2.02** \| **4.83** \| **3.38** \| **2.13** \| **3.99** \| \| 2.0±0.3^a^ \| 1.8±0.24^a^ \| 2.0±0.25^a^ \| 2.1±0.31^a^ \| 3.6±1.02^a^ \| \| 0.7±0.10^a^ \| 0.6±0.08^a^ \| 0.7±0.07^a^ \| 0.8±0.10^a^ \| 1.6±0.46^a^ \| \| 2.0±0.19^ab^ \| 1.3±0.22^b^ \| 1.9±0.04^ab^ \| 2.4±0.22^a^ \| 2.2±0.14^ab^ \| \| **4.76** \| **3.90** \| **4.77** \| **5.42** \| **7.56** \| |

^1^ The amounts are expressed in average of three biological replicates ± SE. Different letters indicate significant difference from pairwise comparison by ANOVA followed by Tukey’s post-hoc test (*p*-value ≤ 0.05).

^2^ DEL-Delphinidins, CYA-Cyanidins, MAL-Malvidins, PEO-Peonidins, PET-Petunidins; GLU-Glucoside, GAL-Galactoside, ARA-Arabinoside.

**Supplementary Table 3**. Concentration polyphenolic compounds (mg g^-1^ DW) quantified by LC-MS in fully ripe bilberries at the end of spectral light treatment from both experimental setups.

|  | **Attached berries**    **Control (W) Control (D) Far-Red Red Blue** | **Detached berries**  **Control (W) Control (D) Far-Red Red Blue** |
| --- | --- | --- |
| **Myr-3-glu**  **Que-3-ara**  **Que-3-rha**  **Syr-3-gal**  **Syr-3-glu**  **Lar-3-gal**  **Lar-3-glu**  **Que-3-gal**  **Que-3-glu**  **Total Flavonols**  **1S-3R**  **Caff-4-glu**  **5Caff-shik**  **Chlor acid**  **Z-Chloro**  **Neo-chlor**  **p-tran.cou**  **Epicatech**  **Catechin**  **Procya B1**  **Procya B2**  **Procya C1**  **Gallocatec**  **Nandin A**  **Leuco-cyanidin** | \| 0.05±0.005^b^ \| \| 0.02±0.004^c^ \| \| \| 0.07±0.004^ab^ \| \| \| 0.09±0.004^a^ \| \| \| 0.05±0.008^bc^ \| \| \| \| --- \| --- \| --- \| --- \| --- \| --- \| --- \| --- \| --- \| --- \| --- \| --- \| --- \| --- \| \| 0.14±0.004^a^ \| \| 0.13±0.029^a^ \| \| \| 0.16±0.001^a^ \| \| \| 0.20±0.023^a^ \| \| \| 0.11±0.013^a^ \| \| \| \| 0.07±0.016^a^ \| \| 0.01±0.007^c^ \| \| \| 0.02±0.001^bc^ \| \| \| 0.05±0.004^ab^ \| \| \| 0.07±0.009^a^ \| \| \| \| 0.10±0.012^a^ \| \| 0.07±0.001^a^ \| \| \| 0.07±0.009^a^ \| \| \| 0.06±0.004^a^ \| \| \| 0.10±0.017^a^ \| \| \| \| 0.01±0.002^a^ \| \| 0.006±0.003^a^ \| \| \| 0.01±0.002^a^ \| \| \| 0.01±0.0006^a^ \| \| \| 0.01±0.002^a^ \| \| \| \| 0.11±0.002^ab^ \| \| 0.05±0.007^b^ \| \| \| 0.06±0.005^b^ \| \| \| 0.10±0.006^ab^ \| \| \| 0.13±0-019^a^ \| \| \| \| 0.02±0.001^ab^ \| \| 0.01±0.0006^b^ \| \| \| 0.02±0.001^ab^ \| \| \| 0.03±0.0005^a^ \| \| \| 0.01±0.003^b^ \| \| \| \| 0.47±0.01^a^ \| 0.52±0.08^a^ \| \| \| 0.53±0.041^a^ \| \| \| 0.64±0.082^a^ \| \| \| 0.37±0.11^a^ \| \| \| \| 0.82±0.031^a^ \| 0.85±0.17^a^ \| \| \| 1.09±0.01^a^ \| \| \| 1.18±0.065^a^ \| \| \| 0.77±0.03^a^ \| \| \| \| **1.8372** \| **1.7025** \| \| \| **2.0817** \| \| \| **2.4021** \| \| \| **1.6617** \| \| \| \| 1.25±0.08^a^ \| \| \| 1.59±0.25^a^ \| \| \| 1.31±0.10^a^ \| \| \| 1.70±0.08^a^ \| \| \| 2.15±0.15^a^ \| \| \| \| 1.28±0.21^b^ \| \| \| 1.78±0.32^ab^ \| \| \| 2.75±0.17^a^ \| \| \| 0.99±0.08^b^ \| \| \| 1.28±0.32^b^ \| \| \| \| 0.13±0.0^bc^ \| \| \| 0.11±0.0^bc^ \| \| \| 0.27±0.02^a^ \| \| \| 0.06±0.001^c^ \| \| \| 0.17±0.021^b^ \| \| \| \| 1.90±0.07^a^ \| \| \| 2.84±0.67^a^ \| \| \| 2.76±0.16^a^ \| \| \| 1.74±0.037^a^ \| \| \| 3.03±0.20^a^ \| \| \| \| 0.12±0.02^a^ \| \| \| 0.25±0.07^a^ \| \| \| 0.27±0.00^a^ \| \| \| 0.1±0.006^a^ \| \| \| 0.14±0.04^a^ \| \| \| \| 0.0±0.00^a^ \| \| \| 0.05±0.02^a^ \| \| \| 5.5±0.00^a^ \| \| \| 0.004±0.00^a^ \| \| \| 0.07±0.0^a^ \| \| \| \| 10.49±0.65^ab^ \| \| \| 4.93±0.77^c^ \| \| \| 12.6±1.0^a^ \| \| \| 7.60±0.76^bc^ \| \| \| 8.61±1.0^abc^ \| \| \| \| 0.56±0.1^a^ \| \| \| 0.96±0.21^a^ \| \| \| 1.0±0.01^a^ \| \| \| 0.76±0.03^a^ \| \| \| 0.53±0.02^a^ \| \| \| \| 0.00±0.0^b^ \| \| \| 0.01±0.0^ab^ \| \| \| 0.01±0.00^ab^ \| \| \| 0.04±0.00^a^ \| \| \| 0.01±0^b^ \| \| \| \| 0.00^a^ \| \| \| 0.0±0.0^a^ \| \| \| 0.03±0.00^a^ \| \| \| 0.01±0.01^a^ \| \| \| 0.007±0^a^ \| \| \| \| 0.88±0.15^b^ \| \| \| 1.41±0.28^ab^ \| \| \| 1.8±0.04^a^ \| \| \| 1.23±0.04^ab^ \| \| \| 1.0±0.32^ab^ \| \| \| \| 0.35±0.06^b^ \| \| \| 0.59±0.16^ab^ \| \| \| 0.83±0.03^a^ \| \| \| 0.48±0.00^ab^ \| \| \| 0.26±0.15^b^ \| \| \| \| 0.01±0.0^b^ \| \| \| 0.00^b^ \| \| \| 0.00±0.0^b^ \| \| \| 0.08±0.00^a^ \| \| \| 0.01±0^b^ \| \| \| \| 0.66±0.06^a^ \| \| \| 1.0±0.1^a^ \| \| \| 0.61±0.0^a^ \| \| \| 0.40±0.02^a^ \| \| \| 0.44±0.05^a^ \| \| \| \| 0.22±0.04^a^ \| \| \| 0.28±0.10^a^ \| \| \| 0.34±0.01^a^ \| \| \| 0.51±0.00^a^ \| \| \| 0. 26±0.23^a^ \| \| \| | \| 0.04±0.004^b^ \| 0.03±0.002^b^ \| \| 0.06±0.002^ab^ \| \| 0.04±0.002^ab^ \| \| 0.07±0.013^a^ \| \| \| --- \| --- \| --- \| --- \| --- \| --- \| --- \| --- \| --- \| \| 0.16±0.001^a^ \| 0.16±0.023^a^ \| \| 0.20±0.028^a^ \| \| 0.19±0.009^a^ \| \| 0.16±0.002^a^ \| \| \| 0.02±0.0012^a^ \| 0.03±0.005^a^ \| \| 0.03±0.007^a^ \| \| 0.03±0.004^a^ \| \| 0.02±0.008^a^ \| \| \| 0.06±0.002^c^ \| 0.19±0.025^a^ \| \| 0.13±0.001^ab^ \| \| 0.07±0.011^bc^ \| \| 0.16±0.005^a^ \| \| \| 0.01±0.001^c^ \| 0.01±0.004^bc^ \| \| 0.02±0.000^ab^ \| \| 0.01±0.002^c^ \| \| 0.02±0.002^a^ \| \| \| 0.04±0.004^b^ \| 0.09±0.011^ab^ \| \| 0.06±0.006^ab^ \| \| 0.04±0.006^ab^ \| \| 0.10±0.026^a^ \| \| \| 0.01±0.002^b^ \| 0.01±0.001^b^ \| \| 0.02±0.000^ab^ \| \| 0.01±0.001^b^ \| \| 0.02±0.005^a^ \| \| \| 0.46±0.050^a^ \| \| 0.51±0.071^a^ \| \| 0.60±0.07^a^ \| \| 0.52±0.033^a^ \| \| 0.48±0.033^a^ \| \| \| 1.45±0.09^ab^ \| \| 1.07±0.021^b^ \| \| 1.30±0.07^ab^ \| \| 1.59±0.063^a^ \| \| 1.10±0.111^b^ \| \| \| **2.2884** \| \| **2.1275** \| \| **2.4605** \| \| **2.5483** \| \| **2.1773** \| \| \| 3.36±0.07^a^ \| \| 2.86±0.08^abc^ \| \| 1.81±0.14^c^ \| \| 3.24±0.50^ab^ \| \| 2.10±0.43^bc^ \| \| \| 0.68±0.0^b^ \| \| 1.97±0.34^a^ \| \| 1.24±0.03^ab^ \| \| 0.67±0.01^b^ \| \| 0.52±0.08^b^ \| \| \| 0.21±0.0^a^ \| \| 0.13±0.01^ab^ \| \| 0.14±0.03^ab^ \| \| 0.21±0.005^a^ \| \| 0.09±0.01^b^ \| \| \| 3.70±0.3^ab^ \| \| 4.23±0.031^s^ \| \| 2.65±0.04^ab^ \| \| 3.68±0.69^ab^ \| \| 2.01±0.70^b^ \| \| \| 0.31±0.0^ab^ \| \| 0.43±0.01^a^ \| \| 0.25±0.01^ab^ \| \| 0.42±0.08^a^ \| \| 0.19±0.06^b^ \| \| \| 0.0 8±0.0^a^ \| \| 0.07±0.01^a^ \| \| 0.04±0.00^a^ \| \| 0.08±0.01^a^ \| \| 0.05±0.0^a^ \| \| \| 12.52±1.47^a^ \| \| 7.37±1.0^b^ \| \| 8.54±0.23^ab^ \| \| 11.9±1.38^ab^ \| \| 7.11±0.92^b^ \| \| \| 1.10±0.04^a^ \| \| 1.0±0.07^a^ \| \| 0.74±0.02^a^ \| \| 1.19±0.02^a^ \| \| 0.74±0.04^a^ \| \| \| 0.03±0.00^a^ \| \| 0.01±0.00^a^ \| \| 0.01±0.00^a^ \| \| 0.03±0.00^a^ \| \| 0.02±0.0^a^ \| \| \| 0.05±0.00^a^ \| \| 0.02±0.00^a^ \| \| 0.03±0.00^a^ \| \| 0.05±0.00^a^ \| \| 0.02±0.01^a^ \| \| \| 1.90±0.05^a^ \| \| 1.76±0.49^a^ \| \| 1.35±0.03^a^ \| \| 1.85±0.05^a^ \| \| 1.20±0.25^a^ \| \| \| 0.86±0.02^a^ \| \| 0.62±0.06^a^ \| \| 0.55±0.00^a^ \| \| 0.77±0.05^a^ \| \| 0.45±0.11^a^ \| \| \| 0.02±0.0^ab^ \| \| 0.00^c^ \| \| 0.005±0.00^bc^ \| \| 0.04±0.00^a^ \| \| 0.03±0.00^a^ \| \| \| 0.91±0.12^b^ \| \| 1.9±0.41^a^ \| \| 1.03±0.03^b^ \| \| 1.13±0.16^ab^ \| \| 0.70±0.06^b^ \| \| \| 0.39±0.05^a^ \| \| 0.41±0.02^a^ \| \| 0.33±0.03^a^ \| \| 0.43±0.03^a^ \| \| 0.50±0.08^a^ \| \| \|  \| \|  \| \|  \| \|  \| \|  \| \| |

^1^ The amounts are expressed in average of three replicates ± SE. Different letters indicate significant difference from pairwise comparison by ANOVA followed by Tukey’s post-hoc test (p-value ≤ 0.05).

^2^ Que-Quercetin, Mye-Myricetin, Lar-Laricitrin, Syr-Syringetin; glu-glucoside/glucuronide, rha-rhamnoside, gal-galactoside, ara-arabinopyranoside. 1S-3R-(1S,3R)-3-(beta-D-glucopyranosyloxy)-1-methylbutyl (2E)-3-(4-hydroxyphenyl) prop-2-enoate), Caff-4-glu-(E)-caffeoyl 4-glucoside, 5caff-shik-5-O-caffeoylshikimic-acid, Chlor acid/Z-Chloro/Neo-chlor-(Z)/neo/Chlorogenic acid, p-tran.cou-p-trans-coumaroyl monotropein, Catechin/Epicatech/Gallocatc -Epicatechin/Gallocatachin, Procya B1/B2/C2 - Procyanidin B1/B2/C2, Nandin-A-Nandinaside A

**Supplementary Table 4**. Details of analytes measured using LC-MS.

| **Compound** | **CAS** | **Exact Mass** | **Formula** | **ID_Confidence^A^** | **RT (min)** | **Equivalence^B^** |
| --- | --- | --- | --- | --- | --- | --- |
| ***Anthocyanins*** |  |  |  |  |  |  |
| cyanidin 3-arabinoside | 27214-72-8 | 419.0973 | C20H19O10 | 2 | 6.9 | cyanidin 3-glucoside |
| cyanidin 3-galactoside | 142506-26-1 | 449.1078 | C21H21O11 | 1 | 5.55 | cyanidin 3-glucoside |
| cyanidin 3-glucoside | 7084-24-4 | 449.1078 | C21H21O11 | 1 | 6.28 | cyanidin 3-glucoside |
| delphinidin 3-arabinoside | 324533-67-7 | 435.0922 | C20H19O11 | 2 | 5.79 | cyanidin 3-glucoside |
| delphinidin 3-galactoside | 28500-00-7 | 465.1028 | C21H21O12 | 2 | 4.5 | cyanidin 3-glucoside |
| delphinidin 3-glucoside | 50986-17-9 | 465.1028 | C21H21O12 | 2 | 5.08 | cyanidin 3-glucoside |
| malvidin 3-arabinoside | 863107-21-5 | 463.1235 | C22H23O11 | 2 | 10.48 | cyanidin 3-glucoside |
| malvidin 3-galactoside | 104880-34-4 | 493.1341 | C23H25O12 | 2 | 9.07 | cyanidin 3-glucoside |
| malvidin 3-glucoside | 7228-78-6 | 493.1341 | C23H25O12 | 1 | 9.71 | cyanidin 3-glucoside |
| peonidin 3-arabinoside | 27214-74-0 | 433.1129 | C21H21O10 | 2 | 9.37 | cyanidin 3-glucoside |
| peonidin 3-galactoside | 260256-26-6 | 463.1235 | C22H23O11 | 2 | 7.94 | cyanidin 3-glucoside |
| peonidin 3-glucoside | 68795-37-9 | 463.1235 | C22H23O11 | 2 | 8.7 | cyanidin 3-glucoside |
| petunidin 3-arabinoside | 749848-37-1 | 449.1078 | C21H20O11 | 2 | 8.2 | cyanidin 3-glucoside |
| petunidin 3-galactoside | 260256-23-3 | 479.119 | C22H23O12 | 2 | 6.84 | cyanidin 3-glucoside |
| petunidin 3-glucoside | 6988-81-4 | 479.119 | C22H23O12 | 2 | 7.47 | cyanidin 3-glucoside |
| ***Polyphenols*** |  |  |  |  |  |  |
| (1S,3R)-3-(beta-D-glucopyranosyloxy)-1-methylbutyl (2E)-3-(4-hydroxyphenyl)prop-2-enoate | 1206159-19-4 | 412.1733 | C20H28O9 | 3 | 6.03 | chlorogenic acid |
| (E)-caffeoyl 4-glucoside | 147511-61-3 | 342.0951 | C15H18O9 | 2 | 2.5 | chlorogenic acid |
| 5-O-caffeoylshikimic acid | 73263-62-4 | 336.0845 | C16H16O8 | 2 | 3.69 | chlorogenic acid |
| catechin | 154-24-4 | 290.079 | C15H14O6 | 1 | 2.69 | catechin |
| chlorogenic acid | 327-97-9 | 354.0951 | C16H18O9 | 1 | 2.74 | chlorogenic acid |
| (Z)-chlorogenic acid | 15016-60-1 | 354.0951 | C16H18O9 | 2 | 3.37 | chlorogenic acid |
| epicatechin | 490-49-0 | 290.079 | C15H14O6 | 1 | 3.39 | epicatechin |
| gallocatechin | 970-73-0 | 306.0739 | C15H14O7 | 2 | 1.45 | epicatechin |
| laricitrin 3-galactoside | 93219-26-2 | 494.106 | C22H22O13 | 2 | 4.84 | quercetin 3-galactoside |
| laricitrin 3-glucuronide | 1026666-46-5 | 508.0853 | C22H20O14 | 2 | 4.85 | quercetin 3-galactoside |
| leucocyanidin | 480-17-1 | 306.074 | C15H14O7 | 2 | 2.23 | epicatechin |
| myricetin 3-galactoside | 15648-86-9 | 480.0904 | C21H20O13 | 2 | 4.1 | quercetin 3-galactoside |
| myricetin 3-glucoside | 19833-12-6 | 480.0904 | C21H20O13 | 2 | 4.18 | quercetin 3-galactoside |
| myricetin 3-glucuronide | 77363-65-6 | 494.0696 | C21H18O14 | 2 | 4.12 | quercetin 3-galactoside |
| nandinaside A | 1813517-24-6 | 446.1213 | C22H22O10 | 3 | 6.27 | epicatechin |
| neochlorogenic acid | 202650-88-2 | 354.0951 | C16H18O9 | 1 | 1.7 | chlorogenic acid |
| p-trans-coumaroyl monotropein | 870785-25-4 | 536.153 | C25H28O13 | 2 | 4.7 | chlorogenic acid |
| procyanidin B1 | 20315-25-7 | 578.1424 | C30H26O12 | 1 | 2.11 | procyanidin B2 |
| procyanidin B2 | 29106-49-8 | 578.1424 | C30H26O12 | 1 | 3.08 | procyanidin B2 |
| procyanidin C1 | 37064-30-5 | 866.2058 | C45H38O18 | 2 | 3.71 | procyanidin B2 |
| quercetin 3-arabinopyranoside | 22255-13-6 | 434.0849 | C20H18O11 | 2 | 5.18 | quercetin 3-galactoside |
| quercetin 3-galactoside | 482-36-0 | 464.0955 | C21H20O12 | 1 | 4.7 | quercetin 3-galactoside |
| quercetin 3-glucuronide | 22688-79-5 | 478.0747 | C21H18O13 | 2 | 4.79 | quercetin 3-galactoside |
| quercetin 3-rhamnoside | 522-12-3 | 448.1006 | C21H20O11 | 1 | 5.37 | quercetin 3-galactoside |
| syringetin 3-galactoside | 55025-56-4 | 508.1217 | C23H24O13 | 2 | 5.44 | quercetin 3-galactoside |
| syringetin 3-glucuronide | 1094607-09-6 | 522.101 | C23H22O14 | 2 | 5.54 | quercetin 3-galactoside |

^A^ Identification confidence: 1 = authentic standard; 2 = accurate mass and supporting information; 3 = accurate and limited supporting information

^B^ Authentic standards were not available for the quantitation of all analytes. In these cases, quantitation was achieved using a chemically similar compound and concentrations are reported as equivalence.
